# Updated national emission inventory and comparison with the Emissions Database for Global Atmospheric Research (EDGAR): case of Lebanon

**DOI:** 10.1007/s11356-021-17562-8

**Published:** 2022-01-08

**Authors:** Anwar Al Shami, Elissar Al Aawar, Abdelkader Baayoun, Najat A. Saliba, Jonilda Kushta, Theodoros Christoudias, Issam Lakkis

**Affiliations:** 1 ALESOPI Consulting, 15 Chemin de Coton, 26120 Chateaudouble, France; 2 Department of Mechanical Engineering, Maroun Semaan Faculty of Engineering and Architecture, American University of Beirut, Beirut, Lebanon; 3 Department of Chemistry, Faculty of Arts and Sciences, American University of Beirut, Beirut, Lebanon; 4 Environmental Predictions Department, Climate and Atmosphere Research Center, The Cyprus Institute, Nicosia, Cyprus

**Keywords:** National inventory, Lebanon, EDGAR inventory, Emissions standard

## Abstract

Physically based computational modeling is an effective tool for estimating and predicting the spatial distribution of pollutant concentrations in complex environments. A detailed and up-to-date emission inventory is one of the most important components of atmospheric modeling and a prerequisite for achieving high model performance. Lebanon lacks an accurate inventory of anthropogenic emission fluxes. In the absence of a clear emission standard and standardized activity datasets in Lebanon, this work serves to fill this gap by presenting the first national effort to develop a national emission inventory by exhaustively quantifying detailed multisector, multi-species pollutant emissions in Lebanon for atmospheric pollutants that are internationally monitored and regulated as relevant to air quality. Following the classification of the Emissions Database for Global Atmospheric Research (EDGAR), we present the methodology followed for each subsector based on its characteristics and types of fuels consumed. The estimated emissions encompass gaseous species (CO, NO_*x*_, SO_2_), and particulate matter (PM_2.5_ and PM_10_). We compare totals per sector obtained from the newly developed national inventory with the international EDGAR inventory and previously published emission inventories for the country for base year 2010 presenting current discrepancies and analyzing their causes. The observed discrepancies highlight the fact that emission inventories, especially for data-scarce settings, are highly sensitive to the activity data and their underlying assumptions, and to the methodology used to estimate the emissions.

**Supplementary Information:**

The online version contains supplementary material available at 10.1007/s11356-021-17562-8.

## Introduction

Poor governments are currently trying to improve air quality by imposing national policies to control pollution sources (Font and Fuller 2016; Jin et al. 2016; Li and Chen 2018). These policies are usually based on the assessment of emission scenarios with the air quality is a severe issue in many countries worldwide affecting public health and ecosystems. In order to effectively manage and mitigate these effects, use of air quality models that investigate the impact of reducing emission rates of the different sources, changing their operating schedule, their technological adaptation or even their spatial distribution. The two main inputs to these models are the driving meteorological conditions, and the inventory of pollutant emissions (Agarwal and Haritash 2014). Forecasting and reanalyzing the meteorology has improved over the years (Yan et al. 2019; Zhao et al. 2016; Ghadban et al. 2020), and having a detailed spatio-temporal emission inventory remains the outstanding major challenge (Bang and Khue 2019; Georgiou et al. 2019). Because they are essential to air quality studies and policy making, global and local emission inventories have been the subject of many studies. Several modules were established and constantly improved to process and compute atmospheric emissions from different sources. Many approaches were proposed to deal with different types of sources and pollutants with varying spatial and temporal resolutions. These efforts aim to build an efficient emissions database with acceptable levels of details for further studies while employing the advances in the meteorological analyses (Guevara et al. 2019, 2020; Grythe et al. 2019). Such modeled estimations can always be verified for precision and accuracy by comparing with measurements and other verified models. This process enables filling gaps and building reliable emission inventories. In addition, monitoring trends of emissions spatially and temporally can serve as a basis for policy making and modification along with subsequent actions that follow (Fameli and Assimakopoulos 2016).

For countries exhibiting scarcity in emissions data, global emission inventories have traditionally been an alternative solution (Madrazo et al. 2018). A well-known and widely used global emission inventory is the Emissions Database for Global Atmospheric Research (EDGAR) developed by the Joint Research Center (JRC). EDGAR provides gridded annual anthropogenic emissions datasets for the period of 1970–2012 on a spatial resolution of 0.1° × 0.1° (Edgar 2019b). The activity data, i.e., fuel consumption for the different sectors is based on international statistics for each country produced by the International Energy Agency (IEA) (Data and statistics 2019). The emission factors utilized in EDGAR datasets are country specific for each sector (Janssens-Maenhout et al. 2015).

EDGAR has been used as a reference to ensure the development of control measures in order to mitigate the effects of certain high emissions (Salameh et al. 2016). It also served as an input to air quality modeling software such as WRF-Polyphemus and WRF-CHEM (Abdallah et al. 2016, 2018; Kedia et al. 2019; Upadhyay et al. 2019). In addition, EDGAR was considered as a validation basis for studies related to emissions of different pollutants using various modeling approaches (Dentener et al. 2002; Bergamaschi et al. 2010). However, a comparison between EDGAR and national developed emission inventories showed that even though EDGAR can be applicable for continental-scale modeling, it is not recommended for regional to local air quality simulations, and a more complete and up-to-date national emission inventory should be used instead (Madrazo et al. 2018; Puliafito et al. 2017; van Amstel et al. 1999; Hristov et al. 2017; Liousse et al. 2019; Georgiou et al. 2020; Kushta et al. 2019).

Lebanon, a small country located on the Eastern coast of the Mediterranean Sea, is considered as a hotspot in the Mediterranean basin and Middle East region in terms of air pollution. Lebanon suffers from an unsustainable road transport sector (Daher et al. 2013; Haddad et al. 2018), unmaintained power plants (Ghanem 2018), and an unregulated private diesel generator sector (Bouri and El Assad 2016; Ghanem 2018; Shihadeh et al. 2018). Therefore, there is a crucial need to conduct accurate air quality modeling studies over the country and in the region (i.e., with reduced uncertainties) based on a refined emission inventory in order to study scenarios and to come up with effective policies to mitigate air pollution impacts. However, the development of national emission inventory is a difficult work to complete since the necessary data such as activity profiles, energy consumption, and fleet characteristics are not always available, even more challenging in a data-scarce environment such as Lebanon.

A complete national emission inventory was first developed for Lebanon for the base year of 2010 by Waked et al. (2012). Later on, a collaboration between the American University of Beirut (AUB) and the Ministry of Environment (MoE) estimated emissions trends for the two key sources of air pollution in Lebanon, diesel generators and light duty vehicles (Baayoun et al. 2019). The study presented a more robust methodology to estimate the emissions than the one adopted by Waked et al. In the current paper, we present a national emission inventory for Lebanon for the base year of 2010 comprising a combination and refinement of the work done by AUB and MoE (Baayoun et al. 2019) and by Waked et al. (2012). We compare, contrast and discuss discrepancies between the newly developed inventory and EDGAR based on total emissions. The base year 2010 was selected because it allows for direct and fair comparison with the previous work done by Waked et al. (2012) which presented an emission inventory for the same year, and with the EDGAR emissions database, which also corresponds to the year 2010. Using the same base year enables us to uniquely and novelly identify discrepancies and for the first time estimate the uncertainties systematically for all pollutants. Our study is further motivated by the outcomes of Salameh et al. (2016) that there is strong evidence that global inventories (including EDGAR) underestimate the emissions by up to a factor of 10 for the transportation sector in Lebanon, and an assessment of anthropogenic emission inventories was deemed necessary as these emissions could be much higher than expected at least from the road transport sector. Emission estimates from light duty vehicles benefit from the use of a more robust methodology than the one adopted by Waked et al. (2012), including emission trends for over a decade, as outlined in Baayoun et al. (2019) for light duty vehicles. For the first time, this work accounts for the emissions of heavy duty vehicles separately leading to a significant revision of previous values in this sector. Also, this study improves markedly upon the previous work by the American University of Beirut (Baayoun et al. 2019) and the Ministry of Environment of Lebanon. Prescribed best practices following EEA standards and recommendations and current state-of-the-art methodology is applied.

This work represents the first national effort in quantifying detailed multisector, multi-species pollutant emissions for Lebanon. The aim is to produce an accurate emission inventory which serves as the backbone of model-based pollution assessment and forecasting, air quality management, policy making, decision making and evaluation of mitigation strategies. This is achieved by following the European Environmental Agency methods, recommendations and practices and using the climate change report by the Ministry of Environment as the main source of activity data (Min 2017; Ministry of Environment 2015a; Ipcc guidelines 2006a). The intercomparison between the three different inventories uncovers and provides an estimate of the associated uncertainties.

## Data and methods

### National inventory

A national emission inventory, including breakdown by sector, was developed for Lebanon. The categorization of the sectors (as shown in Table 1) follows the EDGAR classification and allows for direct intercomparison. We limit the pollutants reported in this study to the ones reported in common by EDGAR and Waked et al. (2012) for the sake of comparison. In the absence of much of the activity data for other pollutants, it was not possible to estimate their emissions. Of the criteria pollutants (SO_*x*_, NO_*x*_, CO, PM, Pb, and O_3_), we did not include O_3_ and Pb. Estimating O_3_ is not meaningful without accounting for its secondary reactions in the atmosphere. As for lead, its emission factors are not available for key sectors. Therefore, our inventory speciation includes emissions of carbon monoxide (CO), nitrogen oxides (NO_*x*_), sulfur dioxide (SO_2_), PM_2.5_ and PM_10_. Emissions per species are estimated using the following formula:

1 $$  Emission = activity\ rate \times emission\ factor $$

**Table 1:** Sectors categorization and type of activity rate for each sector

Sector	Sub	Activity	Emission	Reference
	sectors	rate	factors	
Air transport	Domestic	Energy produced/	IPCC &	This paper
	aviation	fuel consumption	EMEP-EEA	
Ships transport	Domestic navigation	Energy produced/	IPCC &	This paper
		fuel consumption	EMEP-EEA	
Energy	Power plants	Energy produced	EMEP-EEA	This paper
	Diesel generators	Fuel consumption	EMEP-EEA	Baayoun et al. (2019)
Industry	Industrial sites	Fuel consumption	EMEP-EEA	Waked et al. (2012)
Road transport	Light duty	Average traveled	Diesel Net	Baayoun et al. (2019)
	vehicles (LDVs)	distance		
	Heavy duty	Average traveled	Diesel Net/	This paper
	vehicles (HDVs)	distance/fuel		
		consumption/energy	EMEP-EEA/	
		produced	US EPA	
Residential	Space heating	Fuel consumption	EMEP-EEA	This paper
	Cooking	Fuel consumption	EMEP-EEA	This paper
	Agriculture machinery	Fuel consumption	EMEP-EEA	Waked et al. (2012)
	Solid waste	Fuel consumption	EMEP-EEA	Waked et al. (2012)

*IPCC*, Intergovernmental Panel on Climate Change; *EMEP-EEA*, European Monitoring and Evaluation Programme-European Environmental Agency; *US EPA*, United States Environmental Protection Agency

Emission factors used are presented in details in the Supplementary material in Tables S.1 to S.8.

#### Air transport sector

The aviation sector is divided into two categories: domestic aviation and international aviation. Domestic aviation includes flights that depart and arrive at airports within Lebanon and is limited to the operation of five small propeller-type aircraft, used only for training at Beirut International Airport. They usually operate on jet gasoline with an average annual consumption around 3 ktonnes (kt), i.e., 3.30 kt in 2010 (MoE based on personal communication with Cap. Said El Hage in Beirut Airport). The bulk of this sector is represented in the international flights that depart from or arrive at different countries. However, these should be disregarded for the purpose of constructing a national emission inventory (Unf 2019; Aviation emissions and the paris agreement 2016). The air transport sector emissions were estimated based on annual fuel consumption. Average Tier 1 emission factors for CO, SO_2_ and NO_*x*_ for jet gasoline were used based on the IPCC 2006 guidelines (Ipcc guidelines 2006b). The emission factor of PM was retrieved from the EMEP-EEA guidelines (Finstad et al. 2001, 2002). The emission factors obtained from IPCC are in terms of the energy provided by the fuel consumed. As detailed in Table 2, to calculate this energy value, the country-specific net calorific value NCV (provided by the Ministry of Energy and Water, MoEW) of the fuel was multiplied by the annual fuel consumption, which acts as the activity rate. The NCV value used for jet gasoline is 43.50 TJ/kt. Regarding PM (this refers to both *PM*_2.5_ and *PM*_10_ here and elsewhere in this document), the emission factors obtained from EEA are based on the fuel consumption and thus the activity rate used for PM is the annual fuel consumption.

**Table 2:** Sector categorization and type of activity rate for each pollutant in the air transport sector

	**Activity type**	**Activity value**	**Pollutants**	**Emission factors**
**Domestic aviation**	Fuel consumption	3.30 kt	PM	EMEP/EEA (Tier 1)
	Energy produced	143.55 TJ	CO, SO_2_, NO_*x*_	IPCC (Tier 1)

*IPCC*, Intergovernmental Panel on Climate Change; *EMEP-EEA*, European Monitoring and Evaluation Programme-European Environmental Agency

#### Ships transport sector

The covered activities related to shipping include only those related to domestic navigation. These are mainly restricted to fishing boats whose power source is diesel oil (Ministry of Environment 2015b). As detailed in Table 3, sectoral consumption of diesel serving as the activity rate for 2010 was 51.6 kt (Ministry of Environment 2015a). Similarly, as in the air transport section, the Average Tier 1 emission factors for the studied pollutants were retrieved and used in the Tier 1 default approach (EUR 2016b; Eur 2019b).

**Table 3:** Sector categorization and type of activity rate for each pollutant in the ships transport sector (*EMEP-EEA*, European Monitoring and Evaluation Programme-European Environmental Agency)

**Activity type**	**Activity value**	**Pollutants**	**Emission factor**
**Fuel consumption**	51.6 kt	CO, SO_2_, NO_*x*_, PM	EMEP/EEA (Tier 1)
**Energy produced**	1068.6 TJ	CO, SO_2_, NO_*x*_	IPCC (Tier 1)

#### Energy sector

##### Power plants

Annual total energy production data for the seven thermal power plants installed in Lebanon (namely Zouk, Jieh, Hrayche, Deir Ammar, Zahrani, Baalbeck and Tyre) was provided by Ministry of Environment (2015a) as shown in Table 4.

**Table 4:** Annual total energy production from the seven thermal power plants installed in Lebanon

Power plant	Fuel type	Energy (GWh)
Zouk	Heavy fuel oil	2398
Jieh	Heavy fuel oil	1509
Hrayche	Heavy fuel oil	282
Deir Ammar	Diesel oil	2895
Zahrani	Diesel oil	3130
Baalbeck	Diesel oil	201
Tyre	Diesel oil	336

Power plant emissions were estimated using Eq. 1 where the activity rate is the total annual energy produced by the power plants. Since most of these power plants are unmaintained, upper Tier 1 emission factors for fossil fuel power plants were used according to the EMEP/EEA air pollutant emission inventory guidebook (EUR 2016a). The computed activity rates are shown in Section 5.

**Table 5:** Sector categorization and type of activity rate for each pollutant in the energy sector

	**Activity type**	**Activity value**	**Pollutants**	**Emission factor**	**Reference**
**Power plants**					
HFO	Energy produced	2680 GWH	CO, SO_2_, NO_*x*_, PM	EMEP/EEA (Tier 1)	This paper
HFO-5	Energy produced	1509 GWH	CO, SO_2_, NO_*x*_, PM	EMEP/EEA (Tier 1)	This paper
Diesel oil	Energy produced	6562 GWH	CO, SO_2_, NO_*x*_, PM	EMEP/EEA (Tier 1)	This paper
**Diesel generators**	Fuel consumption		CO, SO_2_, NO_*x*_, PM	EMEP/EEA (Tier 2)	Baayoun et al. (2019)

*EMEP-EEA*, European Monitoring and Evaluation Programme-European Environmental Agency

##### Diesel generators

Baayoun et al. (2019) estimated the annual emissions from diesel generators in Lebanon spanning the period 2009–2016. Baayoun et al. (2019) The total amount of fuel consumed by diesel generators in Lebanon was derived from two sources: the first was the total amount of fuel imported and reported by the Central Administration of Statistics (CAS) (Cas statistical yearbook 2016) on behalf of the MoEW and the second was the distribution by sector as stated by one of the major oil and gas companies, Issa Petrol Trade Energy Center (IPTEC) (MoE, 2017; shared source data). Table 5 lists the energy sector categories and type of activity rate for each pollutant. Diesel generator emissions were calculated according to the EMEP/EEA air pollutant emission inventory guidebook using the Tier 2 methodology for reciprocating engine applications and the emission factors found in the small combustion data of the guidebook (EUR 2016b).

#### Industry

The emissions of this sector were estimated by Waked et al. (2012) based on the total fuel consumed by the different Lebanese industries. These quantities are reported based on the CAS, MoEW statistics of industrial fuel imports for 2010 (Cas statistical yearbook 2016). The activity rate is equivalent to 199.724 kt of fuel oil. This quantity is then associated with their relative Tier 2 technology specific emission factors (Eur 2019a).

#### Road transport

The estimation of the traffic emissions takes into account the characteristics of the vehicle fleet (the model type, model year, fuel type used, origin, number of cylinders, energy output of the motor, and the year of circulation). The database for these characteristics was obtained from the national vehicle registry (Min 2017) for the base year 2010. Vehicles were classified into two categories: light duty vehicles (LDVs) and heavy duty vehicles (HDVs). Any vehicle with a weight less than 3,500 kg is referred to an LDV; otherwise, it is considered as an HDV.

##### Light duty vehicles

Analyzing the fleet distribution, LDVs constitute the majority of the circulating vehicles for each year at around 90% of the total fleet during 2010. The LDVs were further classified based on the origin of each vehicle into three groups: European origin (EU), US origin (US) and Japanese origin (Jap) with Korean origin vehicles being included in the Japanese ones as they follow the Japanese standards. The activity rate used in retrieving the emissions of this sector is the total annual traveled distance of all LDVs. The average traveled distance by vehicle origin was obtained using vehicle fuel efficiencies and national fuel consumption (Baayoun et al. 2019).

In addition, average Tier 2 emission factors were obtained for all pollutants, except SO_2_, based on the type of fuel consumed (gasoline or diesel) according to the three groups of different origins of LDVs based on the categorization of Dieselnet (2019). The SO_2_ emission factor was calculated based only on the sulfur content of the fuel consumed, equivalent to Tier 1 emissions related to annual fuel consumption.

2 $$  E_{SO_{2}}= k_{s,m} \times FC $$

where E_*SO*2_ = emission of sulfur dioxide (in Gg), k_*s*,*m*_= sulfur mass content in fuel m (in Gg/ tons of fuel), and FC= fuel consumption (in tons). Activity rates are summarized in Table 7.

##### Heavy duty vehicles

Similarly, the total fleet distribution was filtered to get the total HDVs circulating in 2010. These were also divided based on the origin of each vehicle into three groups: EU origin, US origin and Asian origin. Furthermore, each group was divided into two subgroups based on the type of fuel consumed (diesel or gasoline “Commercial Trucks” DieselNet 2019) as shown in Table 6.

**Table 6:** Heavy duty vehicle (HDV) fleet classification per origin and fuel type (total numbers for 2010)

HDV	EU origin	US origin	Asian origin
Diesel	10,883	668	17,914
Gasoline	29,285	4,132	45,045

**Table 7:** Sector categorization and type of activity rate for each pollutant in the road transport sector. *EMEP-EEA*, European Monitoring and Evaluation Programme-European Environmental Agency

	**Activity type**	**Activity value**	**Pollutants**	**Emission factor**	Reference
**Light duty vehicles (< 3.5 ton)**					Baayoun (2019)
Gasoline	Average distance traveled	2.40E10 km	CO, NO_*x*_, PM	DieselNet (2019)	
Gasoline	Fuel consumption		SO_2_	General Directorate of Oil (Baayoun et al. 2019)	
Diesel	Average distance traveled	5.28E10 km	CO, NO_*x*_, PM	DieselNet (2019)	
Diesel	Fuel consumption		SO_2_	General Directorate of Oil (Baayoun et al. 2019)	
**Heavy duty vehicles**					This paper
EU origin Diesel	Average distance traveled	5.44E08 km	CO, NO_*x*_, PM	EMEP/EEA (Tier 2) (UNECE 2011)	
EU origin Gasoline	Average distance traveled	7.98E08 km	CO, NO_*x*_, PM	EMEP/EEA (Tier 2) (UNECE 2011)	
US origin Diesel	Average distance traveled	0.33E08 km	CO, NO_*x*_, PM	EMEP/EEA (Tier 2) (Reichle et al. 2015)	
US origin Gasoline	Average distance traveled	1.13E08 km	CO, NO_*x*_, PM	EMEP/EEA (Tier 2) (Reichle et al. 2015)	
Asia origin Diesel	Average distance traveled	8.96E08 km	CO, NO_*x*_, PM	EMEP/EEA (Tier 2) (Dieselnet 2019)	
Asia origin Gasoline	Average distance traveled	12.27E08 km	CO, NO_*x*_, PM	EMEP/EEA (Tier 2) (Dieselnet 2019)	
EU origin Diesel	Fuel consumption	130.60 kt	SO_2_	EMEP/EEA (Tier 1) (UNECE 2011)	
EU origin Gasoline	Fuel consumption	79.80 kt	SO_2_	EMEP/EEA (Tier 1) (UNECE 2011)	
US origin Diesel	Fuel consumption	8.02 kt	SO_2_	DieselNet (Dieselnet 2019)	
US origin Gasoline	Fuel consumption	11.26 kt	SO_2_	DieselNet (Dieselnet 2019)	
Asia origin Diesel	Fuel consumption	214.97 kt	SO_2_	EMEP/EEA (Tier 1) (Dieselnet 2019)	
Asia origin Gasoline	Fuel consumption	122.75 kt	SO_2_	EMEP/EEA (Tier 1) (Dieselnet 2019)	

The emission factors for each subgroup were obtained as follows: average Tier 2 emission factors were used for all US origin HDVs (Reichle et al. 2015) and Asia Gasoline HDVs (Dieselnet 2019). The activity rate is the annual total traveled distance which is 50,000 km/vehicle in case of diesel HDVs and 27,250 km/vehicle in case of gasoline HDVs. These traveled distances are obtained using the ForFITS (For Future Inland Transport Systems) model which uses transport data provided by national and international transport agencies. Data for Lebanon was obtained using the model for countries with similar mobility characteristics (UNECE 2011). As for EU origin vehicles, average Tier 1 emission factors were used to estimate their emissions based on the annual fuel consumption (EUR 2016b).

Since the activity rate for the HDVs is the traveled distance, the estimation of the annual fuel consumption was made using the average consumption of each fuel per unit of distance traveled. That is on average 240 g/km for diesel HDVs and 100 g/km for gasoline HDVs as per EMEP/EEA. For the last subgroup, the Asian origin diesel HDVs, emission factors are based on the total energy produced (Dieselnet 2019). The energy produced is obtained based on the motor specifications provided by the national fleet registry documents. All the above specification refer to emission factors for CO, NO_*x*_, PM_2.5_ and PM_10_. SO_2_ emission factors are based on the sulfur content of the fuel consumed and are always referred to as Tier 1 emissions. Equation 2 was used to calculate the SO_2_ emission factors for European HDVs (EUR 2016b) and Eastern HDVs, as these generally followed EURO III standards in 2010 (Wang et al. 2014). US HDVs SO_2_ emission factors were obtained from DieselNet (Dieselnet 2019).

#### Residential sector

##### Space heating and cooking

The major types of fuel used for heating in Lebanon are gasoil, liquefied petroleum gas (LPG), and wood. Since no accurate data on the share of LPG and wood quantity used in this sector are available for analysis (UNFCCC 2011), we only consider the emissions due to residential boilers relying on gasoil. Fuel consumption data of this latter was obtained from IPTEC (MoE, 2017). In total, 172,249 metric tons (MT) of gasoil were estimated to be used for space heating in 2010. A net calorific value of 43.38 GJ/tons was used for gasoil. Space heating emission factors were taken according to the EMEP/EEA air pollutant emission inventory guidebook using the Tier 2 methodology for small boiler (residential) applications (EUR 2016b). The commonly used cooking appliances in Lebanon are predominantly gas stoves. Fuel consumption data of LPG used by these cooking appliances was obtained from IPTEC (MoE, 2017). In total, 177,858 MT of LPG were used for cooking in 2010. Emission factors were taken according to EMEP/EEA air pollutant emission inventory guidebook using the Tier 2 methodology for stoves burning gas (EUR 2016b). The calorific value of 50 GJ/tons was used for LPG.

##### Agriculture and solid waste

Waked et al. estimated the emissions of this sector in an approach similar to that used in the industry sector (Waked et al. 2012) that is based on the total fuel consumed by the agriculture machinery and the relative masses of the different types of incinerated wastes for 2010. These quantities are then associated with their relative Tier 1 emission factors as advised by EMEP-EEA guidelines (EUR 2016b).

### EDGAR HTAP equivalents

Yearly values by sector for 2010 were obtained from the HTAP_V2 inventory dataset compiled by EDGAR team (JRC) (Edgar 2019a). HTAP_V2 is a compilation of different regional gridded inventories including that of EPA for the USA, the EPA and Environment Canada (for Canada), the EMEP and Netherlands Organisation for Applied Scientific Research (TNO) for Europe, and the Model Intercomparison Study for Asia (MICS-Asia III) for China, India and other Asian countries, while the rest of the world emissions are complemented by EDGARv4.3 (Janssens-Maenhout et al. 2015). The HTAP emission inventory provides data as emission fluxes (kg/m^2^/s). Yearly emissions were calculated and summed over Lebanon. The implied emission factors per sector used by EDGAR for Lebanon are given in Table 8.

**Table 8:** Implied emission factors (in g/GJ) for Lebanon per species per source sector for 2010 from EDGAR HTAP_v2

	CO	NO_*x*_	SO_2_	PM_2.5_	PM_10_
Energy	123.73	617.32	1113.72	6.65	8.95
Industry	52.88	150.50	691.88	56.08	76.48
Residential	345.56	67.89	155.84	26.82	50.23
Transport	1301.31	62.94	11.98	2.21	2.21

## Results and discussion

### Updated national inventory

**Fig. 1:**
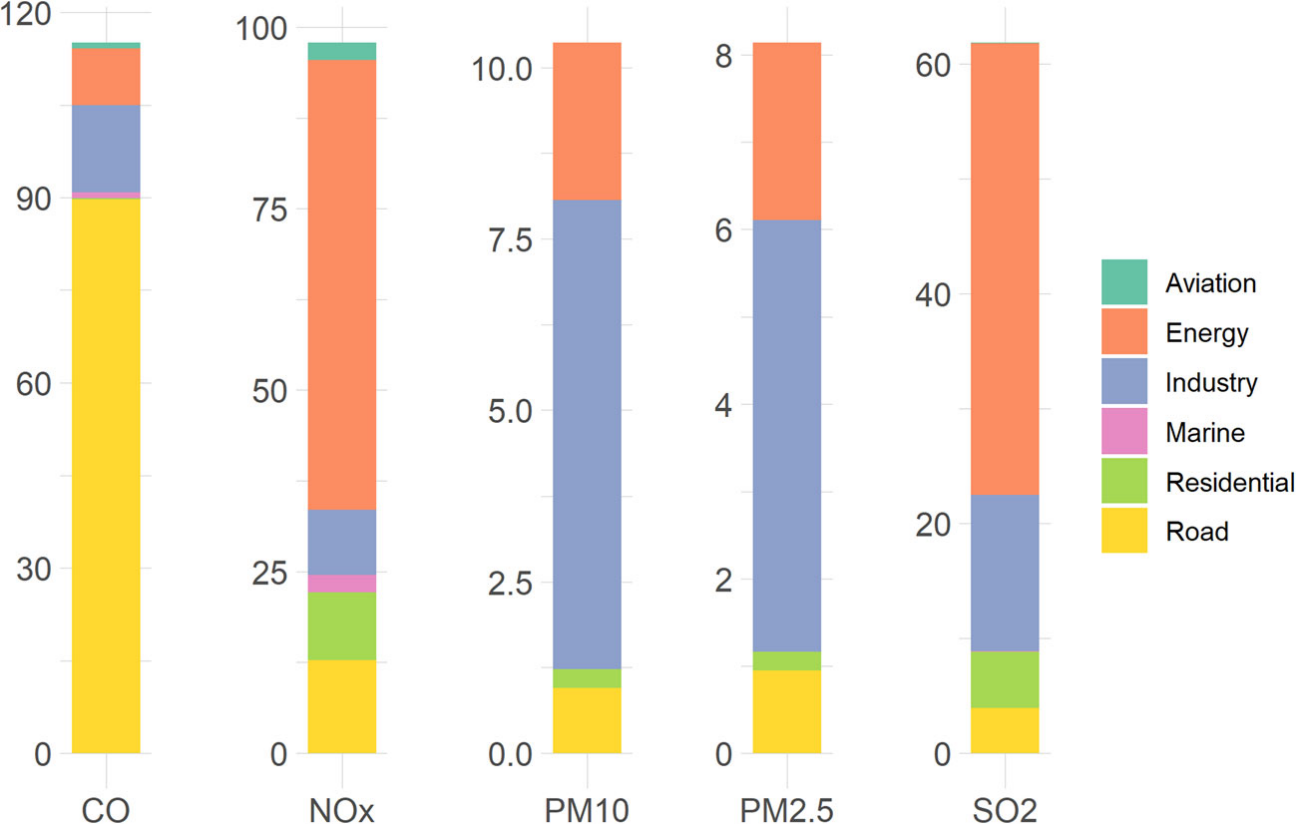
Emissions of the different pollutants by sector (in Gg)

The total estimated emissions by source sector for the year 2010 for CO, NO_*x*_, SO_2_, PM_2.5_ and PM_10_ are 115.37, 97.31, 63.82, 8.15 and 10.36 Gg respectively. The contribution of each sector to the total emissions of each pollutant is shown in Fig. 1. We note that road transport is the major sector contributing to CO emissions with a percentage of 77.7%. Most CO emissions within this sector are mainly due to the LDV subsector (only gasoline vehicles as per Lebanese regulations) which alone releases 57% of the total CO emissions. The second contributor is the HDV sector (mostly diesel vehicles) contributing around 31.7% of the total CO emissions. As for NO_*x*_ and SO_2_ emissions, the bulk of these pollutants originate from the energy sector. The energy sector emits 63.6% of total NO_*x*_ and diesel generators alone contribute 52.79 Gg of NO_*x*_. Energy production also emits 61.6% of total SO_2_ where the major contribution of 36.62 Gg is from the power plants subsector. The industry sector estimations indicate that this sector is the predominant major polluting source for PM_2.5_ and PM_10_ with percentages of 60.81% and 66.4% respectively. The residential sector was found to be a small contributor across all pollutants as the only sources that could be estimated (as per EDGAR sector categorization) given data availability were the solid waste and agricultural machinery subsectors. The Agriculture sector was not estimated due to the absence of data. Among all the covered sectors and subsectors, we notice that the LDVs and diesel generators subsectors are the major contributors to total emissions of all the studied pollutants with values of 66.89 Gg and 66.13 Gg respectively. That is due to the fact that the most dominant mode of transport in Lebanon is passenger cars with a ratio of 1 cars every 3 individuals (Chaaban et al. 2001). The random unregulated growth of the diesel power generation private sector is an emerging phenomenon in the Lebanese community caused by and compensating for the national electricity shortage.

### Comparison between different emission inventories for Lebanon

**Fig. 2:**
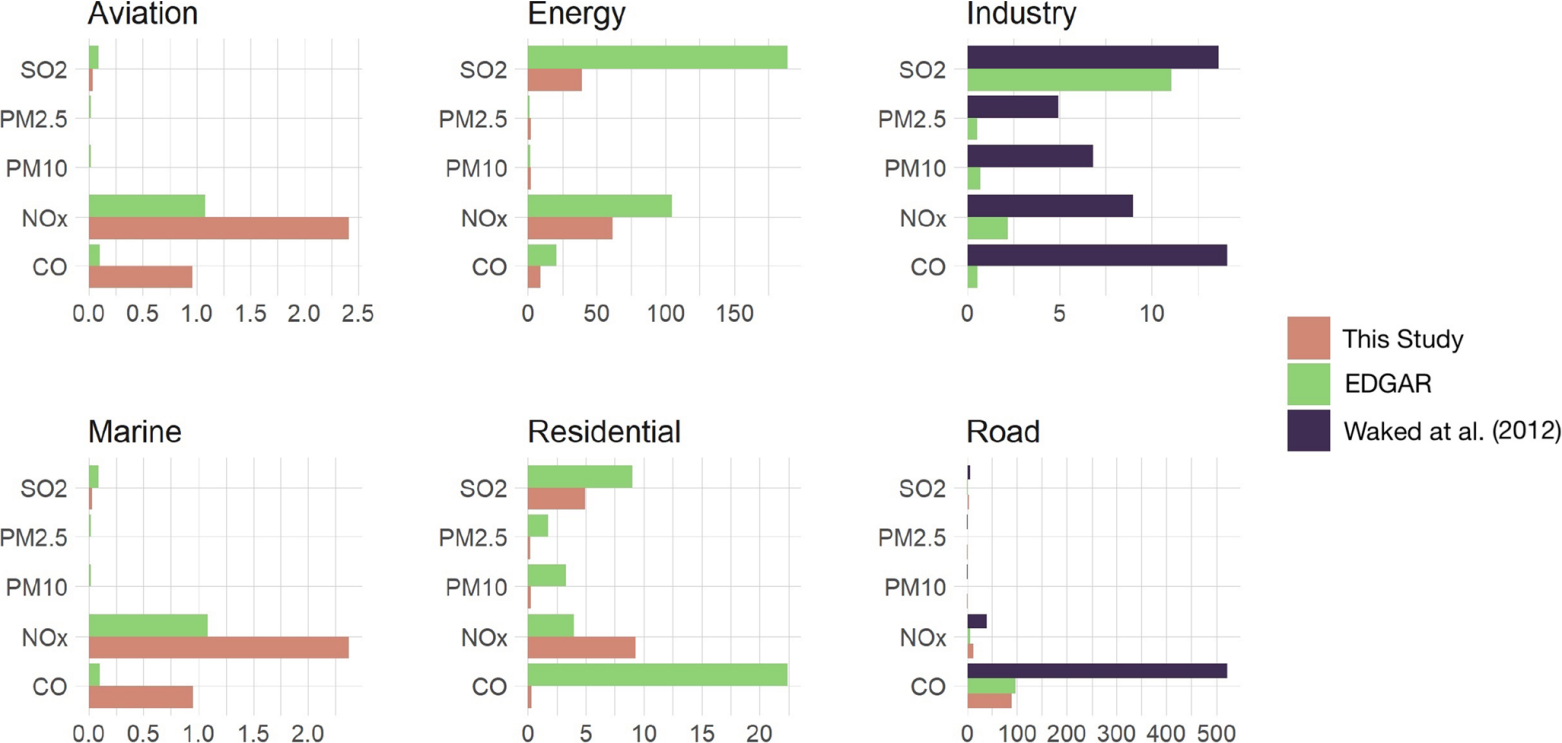
Comparison of emissions (in Gg) per pollutants per sector based on our national developed emission inventory

The total of each studied sector is compared to that of the corresponding sector in EDGAR emission inventory. Our results were compared, specifically for the road sector, with those of Waked et al. (2012). The reason why that the comparison was made for this specific sector solely is that Waked et al. (2012) do not state the emissions of other subsectors, such as air and marine transport. In addition, the sub-divisions of the energy and residential sectors between the newly developed and Waked et al. inventory are different. Our inventory considers the diesel generators as a subsector of the energy sector, while Waked et al. considers it to be a part of the residential sector. The categorization we adopted is largely based on EDGAR classification. One notable difference is that while EDGAR accounts for emissions data on international activities in the air and marine transport sectors, we are dealing with a national-scale emission inventory and thus, we only considered emissions coming from national aviation and domestic navigation restricted to fishing boats. For the industry sector, we have used the same systematic approach to deal with the different industries in Lebanon as reported in Waked et al. Intercomparison results are shown in Fig. 2.

For both the air and marine transport sectors, our emission estimates are higher than those of EDGAR by a factor of 9 in the case of CO and by a factor of 2 for NO_*x*_, and lower by a factor of 3 for SO_2_, PM_2.5_ and PM_10_. As for the energy sector, EDGAR emissions are higher than our estimated values by a factor of 2 for CO and NO_*x*_ and by a factor of 5 for SO_2_; yet, the EDGAR totals amount to about half our estimate for PM_2.5_ and PM_1_0. This can be attributed to the emission factors used in EDGAR inventory (Janssens-Maenhout et al. 2015) (EDGAR applies a uniform emission factor for all the energy sector in Lebanon). Our methodology, however, uses different emission factors for the different types of fuels used in each subsector as shown in Section ??. For the inventories considered, a large difference in estimated emissions is also found for the industry sector by Waked et al. The basic difference in methodology for the emission factors can be seen in Table 8. Uniform emission factors are used for the industry sector as a whole whereas Waked uses a technology or industry specific emission factor in each category of industries present in Lebanon. The same applies for the observed discrepancies at the level of the residential sector as our estimated emissions are about twice those estimated in EDGAR for the case of NO_*x*_ only and lower for other studied pollutants as shown in Fig. 2.

**Fig. 3:**
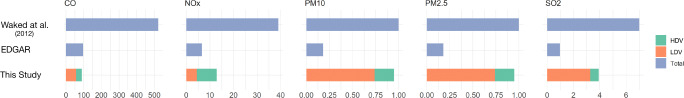
Comparison of emissions (in Gg) of the road transport sector per pollutant

The road transport sector emissions obtained are compared with both EDGAR and Waked inventory in Fig. 2. Compared to Waked et al., a significant difference in CO and NO_*x*_ emissions is observed; the estimated emissions in Waked et. al are around 5 times higher. This is attributed to the fact that Waked used the EEA-EMEP emission factors for their estimations (Waked et al. 2012) which implies that all the vehicle fleet is assumed to be from European origin. The emission factors of EEA are usually greater than those for different standards. Our methodology classified the vehicle fleet per origin and used the corresponding emission factors. A comparison of the road transport sector results for all pollutants is depicted in Fig. 3.

**Fig. 4:**
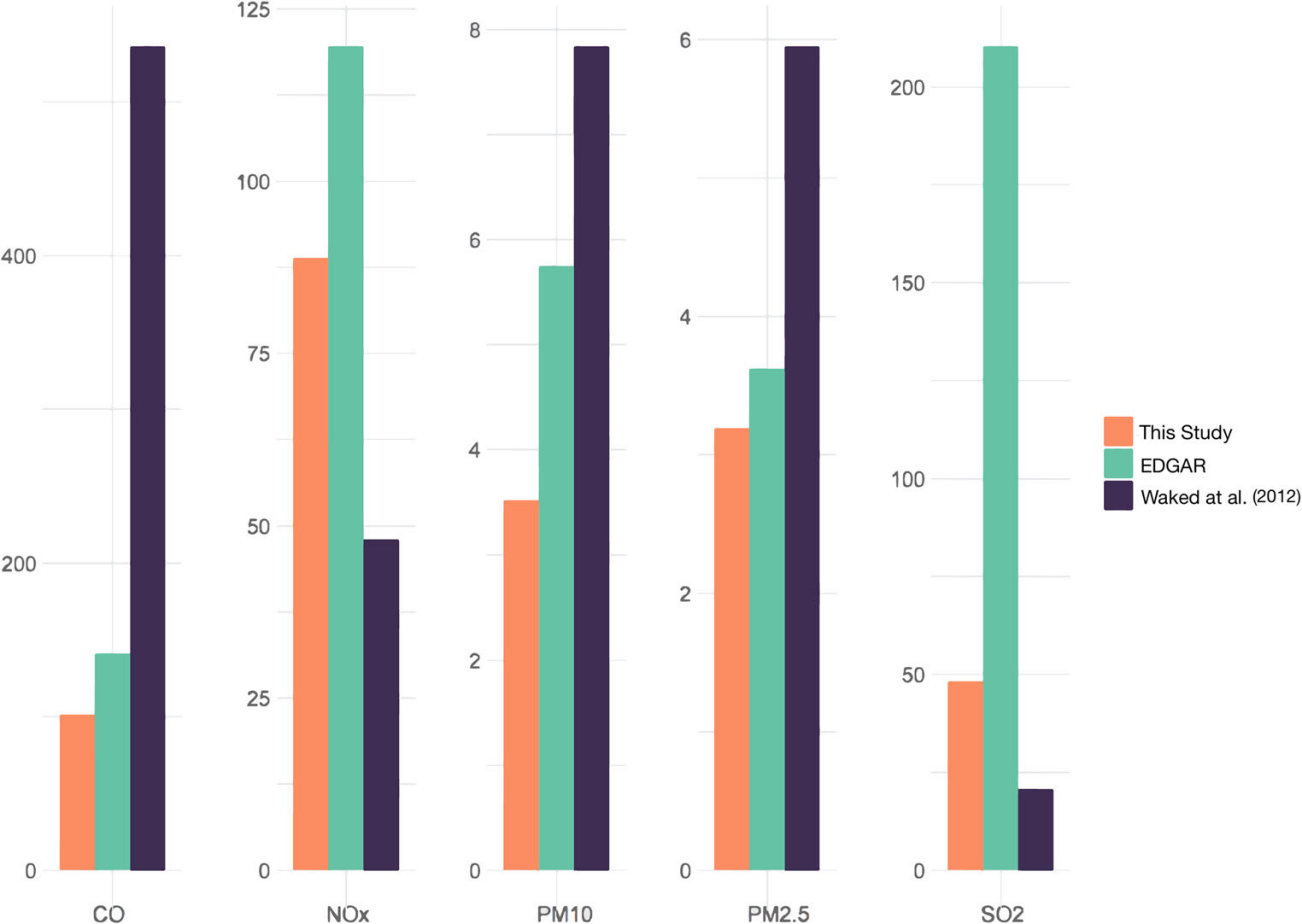
Comparison of total emissions (Gg) of all studied sectors between our national inventory, the EDGAR inventory and the Waked et al. (2012) inventory

Similarly, EDGAR used uniform emission factors throughout the transport sector and its subsectors and for all fuels used as indicated in Table 8. Our national inventory estimations for the road transport sector are within the range of values from EDGAR and Waked inventory for all the studied pollutants, with particulate matter totals closer to Waked et al. estimations. We also note that our total estimated emissions for NO_*x*_ and SO_2_ for all sectors are higher than the estimations of Waked et al. (2012) and lower than EDGAR, while for CO and PMs, we obtain lower annual totals from both other inventories as can be seen in Fig. 4. There is considerable difference between our total estimated CO emissions and those in Waked et. al. There are indications that the latter may have overestimated the CO emissions. An air pollution modeling study (Waked et al. 2013) from 2 to 18 July 2011 using WRF-Polyphemus with the emission inventory reported in Waked et. al. as an input reported mean modeled concentration values over-predicted, by about 30% on average, the observations from a monitoring station located at the University of Saint Joseph (USJ), Beirut. We note however that the data are based on observations from a single monitoring station, and for a small time period (16 days) and may not be representative for the whole country. Based on this study, additional modelling and observation efforts are needed to reach well-founded conclusions on this matter.

## Conclusions and outlook

We presented an updated national emissions inventory for Lebanon for atmospheric pollutants that are internationally monitored and regulated as relevant to air quality. By exhaustively quantifying detailed multisector, multi-species pollutant emissions, this work serves as a stride to fill the gap by the absence of a clear emission standard and standardized activity datasets in Lebanon. To this end, several methods and input data for the year 2010 were used for the different sectors and subsectors and based on the various types of fuels consumed to refine the accuracy of the estimates.

The updated inventory was compared with EDGAR emission totals for the year 2010 for all sectors and species with several differences emerging. In addition, the road transport subsector was compared with both EDGAR and Waked et al. (2012) inventories where remarkable differences were also apparent. The emissions reported in this paper supersede previously published estimates (Waked et al. 2012) by including emissions for key sources of air pollution in Lebanon that were previously unaccounted for: diesel generators (a major source of energy production and pollution), air and ship transport, and residential space heating and cooking. A large difference in the estimated emissions is also found for the industry sector. This work also improved on previous assumptions (both in waked et al. 2010 and in EDGAR) and used a more detailed analysis of activity data. Major efforts were placed in treating and refining the data, hence revealing how differently that contributed to major discrepancies in the final results of the inventories. The data gaps and discrepancies found highlight the following:

- In Lebanon, the road transport, especially LDVs, and diesel generators subsectors are the major contributors to total emissions of all the studied pollutants.
- For the road transport sector, the estimated CO and NO_*x*_ emissions in Waked et al. (2012) are around 5 times higher than our estimates. For the same sector, the estimated EDGAR emissions of PM2.5 and PM10, NO_*x*_, and SO_*x*_ are around 5, 2, and 5 times lower than our estimates.
- Total estimated emissions for NO_*x*_ and SO_2_ for all sectors are higher than the estimations of Waked et al. (2012) and lower than EDGAR, while CO and PMs annual totals are lower than both other inventories.
- The activity data and their underlying assumptions lead to significant differences in the estimated emissions.
- Emission estimates strongly depend on the methodology and emission factors used, as observed in the estimates for CO in the industry sector and road, air, and marine transport sectors, SO_2_ for the energy sector, NO_*x*_ for the residential, industry, and marine and road transport sectors, and PMs in the industry and residential sectors.

The observations reveal that emission inventories, especially for data-scarce settings, have a double sensitivity: the activity data and their underlying assumptions lead to significant differences in the estimated emissions on the one hand, and, on the other hand, emission estimates vary significantly depending on the methodology used. As such, in the absence of a clear emission standard and standardized activity datasets in Lebanon, future work will focus on further improving the accuracy of the input data required for the estimations. On the other hand, it is of great importance to obtain an inventory taking into account the spatial and temporal distribution of the emissions of the several pollutants in Lebanon.

## Electronic supplementary material

Below is the link to the electronic supplementary material.

(PDF 102 KB)
