# Supplementary material for: Updated national emission inventory and comparison with the Emissions Database for Global Atmospheric Research (EDGAR): case of Lebanon
Source: Environ Sci Pollut Res Int. 2022 Jan 8;29(20):30193–205. doi: 10.1007/s11356-021-17562-8 (PMC9001561; doi:10.1007/s11356-021-17562-8)
Supplement: Supplementary file 1 — (PDF 102 KB) [file 11356_2021_17562_MOESM1_ESM.pdf]

Noname manuscript No.
(will be inserted by the editor)

Updated national emission inventory and comparison with the Emissions Database for Global Atmospheric Research (EDGAR): Case of Lebanon

Anwar Al Shami · Elissar Al Aawar · Abdelkader Baayoun · Najat A. Saliba · Jonilda Kushta · Theodoros Christoudias · Issam Lakkis

Received: date / Accepted: date

Anwar Al Shami
ALESOPI Consulting
15 Chemin de Coton
26120 Chateaudouble, France
E-mail: anwar.alshami@alesopi.com

Elissar Al Aawar
Department of Mechanical Engineering
Maroun Semaan Faculty of Engineering and Architecture
American University of Beirut
Beirut, Lebanon
E-mail: eta10@mail.aub.edu

Abdelkader Baayoun
Department of Mechanical Engineering
Maroun Semaan Faculty of Engineering and Architecture
American University of Beirut
Beirut, Lebanon
E-mail: ahb25@mail.aub.edu

Najat A. Saliba
Department of Chemistry
Faculty of Arts and Sciences
American University of Beirut
Beirut, Lebanon
E-mail: ns30@aub.edu.lb

Jonilda Kushta
Environmental Predictions Department
Climate and Atmosphere Research Center
The Cyprus Institute
Nicosia , Cyprus
E-mail: j.kushta@cyi.ac.cy

Theodoros Christoudias
Environmental Predictions Department
Climate and Atmosphere Research Center
The Cyprus Institute
Nicosia , Cyprus
E-mail: t.christoudias@cyi.ac.cy

1 Supplementary Material

In this supplement, we present the different used emission factors for the studied pollutants in the sectors.

1.1 Air transport Sector Emission Factors

The emission factors for all studied species represented in Table S.1 are the Average Tier 1 emission factors for jet gasoline used for domestic aviation.

1.2 Marine transport Sector Emission Factors

The emission factors for all species represented in Table S.2 are the Average Tier 1 emission factors for diesel oil used in marine aviation.

1.3 Power Plant Subsector Emission Factors

The emission factors for all species represented in Table S.3 are the upper Tier 1 emission factors for the two types of fuel used in energy production.

1.4 Diesel Generators Subsector Emission Factors

The emission factors for all species presented in Table S.4 are the Tier 2 emission factors for diesel generators.

1.5 Light Duty Vehicles Subsector Emission Factors

The emission factors for all species are averaged emission factors for the LDVs fleet of 2010 are presented in Table S.5.

Issam Lakkis
Department of Mechanical Engineering
Maroun Semaan Faculty of Engineering and Architecture
American University of Beirut
Beirut, Lebanon
E-mail: il01@mail.aub.edu

	CO(kg/TJ)	NO_x (kg/TJ)	SO_2 (kg/kt)	$PM_{2.5}$ (kg/TJ)	PM_{10} (kg/TJ)
Jet gasoline	100	250	43.75	64	64

Table S.1: Average Tier 1 emission factors used for the air transport sector.

	CO	NO_x	SO_2	$PM_{2.5}$	PM_{10}
Diesel oil	7.4	78.5	20	1.4	1.5

Table S.2: Tier 1 emission factors used for the marine transport sector in kg/ton fuel.

	CO	NO_x	SO_2	$PM_{2.5}$	PM_{10}
Diesel oil	65	195	465	2.5	10
Heavy fuel oil	21.1	300	1700	90	150

Table S.3: Upper Tier 1 emission factors used for the power plant subsector in g/GJ.

	CO	NO_x	SO_2	$PM_{2.5}$	PM_{10}
Diesel oil	130	942	48	30	30

Table S.4: Tier 2 emission factors used for the diesel generators subsector in g/GJ.

	CO	NO_x	$PM_{2.5}$	PM_{10}
Gasoline	1.8	0.12	0.021	0.021

Table S.5: Averaged emission factors used for the LDV fleet subsector in g/km.

1.6 Heavy Duty Vehicles Subsector Emission Factors

The emission factors for all species presented in Table S.6 are the various emission factors for for the different categories of HDVs.

	CO	NO_x	SO_2	$PM_{2.5}$	PM_{10}
EU diesel	7.58 g/kg fuel	33.37 g/kg fuel	0.006 g/kg fuel	0.94 g/kg fuel	0.94 g/kg fuel
EU gasoline	152.3 g/kg fuel	13.22 g/kg fuel	0.01 g/kg fuel	0.02 g/kg fuel	0.02 g/kg fuel
US diesel	8.1 g/mile	0.9 g/mile	0.015 g/ kg fuel	0.02 g/mile	0.02 g/mile
US gasoline	7.3 g/mile	1.53 g/mile	0.084 g/kg fuel	0.012 g/mile	0.012 g/mile
Asia diesel	2.22 g/kWh	0.4 g/kWh	0.08 g/kg fuel	0.01 g/kWh	0.01 g/kWh
Asia gas.	2.55 g/km	0.07 g/km	0.08 g/kg fuel	0.07 g/km	0.07 g/km

Table S.6: Different emission factors used for the HDV subsector.

1.7 Space Heating Subsector Emission Factors

The emission factors for all species represented in Table S.7 are Tier 1 emission factors for the space heating subsector.

	CO	NO_x	SO_2	$PM_{2.5}$	PM_{10}
Gasoil	3.7	69	79	1.5	1.5

Table S.7: Tier 1 emission factors used for the space heating subsector in g/GJ.

1.8 Cooking Subsector Emission Factors

The emission factors for all species represented in Table S.8 are Tier 1 emission factors for the cooking subsector.

	CO	NO_x	SO_2	$PM_{2.5}$	PM_{10}
LPG	30	60	0.3	2.2	2.2

Table S.8: Tier 1 emission factors used for the cooking subsector in g/GJ.
